# Endogenous antioxidants predicted outcome and increased after treatment: A benzoate dose‐finding, randomized, double‐blind, placebo‐controlled trial for Alzheimer's disease

**DOI:** 10.1111/pcn.13504

**Published:** 2022-11-24

**Authors:** Hsien‐Yuan Lane, Shi‐Heng Wang, Chieh‐Hsin Lin

**Affiliations:** ^1^ Department of Psychiatry & Brain Disease Research Center China Medical University Hospital Taichung Taiwan; ^2^ Graduate Institute of Biomedical Sciences China Medical University Taichung Taiwan; ^3^ Department of Psychology, College of Medical and Health Sciences Asia University Taichung Taiwan; ^4^ Department of Occupational Safety and Health China Medical University Taichung Taiwan; ^5^ Department of Psychiatry, Kaohsiung Chang Gung Memorial Hospital Chang Gung University College of Medicine Kaohsiung Taiwan; ^6^ School of Medicine Chang Gung University Taoyuan Taiwan

**Keywords:** Alzheimer's disease, antioxidants, biomarkers, dementia, treatment response

## Abstract

**Aim:**

Previous pilot studies suggest that sodium benzoate may be a potential cognitive enhancer for patients with Alzheimer's disease (AD), schizophrenia, or late‐life depression. Especially for AD treatment, a confirmatory trial with predictive biomarkers is urgently needed. This study aimed to confirm benzoate as a novel treatment for AD and to discover its optimal dose and biomarkers.

**Methods:**

A 24‐week, dose‐finding, randomized, double‐blind, placebo‐controlled trial, with clinical measurements at weeks 0, 8, 16, and 24, was conducted in three major medical centers in Taiwan. Among 154 patients screened for AD, 149 were eligible and randomized to one of the four treatments: (i) benzoate 500 group (fixed 500 mg/day); (ii) benzoate 750 (500 mg/day for the first 4 weeks, 750 mg/day from the 5th week); (iii) benzoate 1000 (500 mg/day for the first 4 weeks, 1000 mg/day from the 5th week); and (iv) placebo. The primary outcome measure was AD assessment scale‐cognitive subscale (ADAS‐cog).

**Results:**

The benzoate 1000 group performed best in improving ADAS‐cog (*P* = 0.026 at week 24), with female advantage. Higher plasma catalase at baseline predicted better outcome. Benzoate receivers tended to have higher catalase and glutathione than placebo recipients after treatment. The four intervention groups showed similar safety profiles.

**Conclusions:**

By enhancing two vital endogenous antioxidants, catalase and glutathione, sodium benzoate therapy improved cognition of patients with AD, with higher baseline catalase predicting better response. Supporting the oxidative stress theory, the results show promise for benzoate as a novel treatment for AD.

Dementia is gaining increasing attention because of the high mortality and morbidity, which needs collaborative care.^1^ Early intervention is the key for dementia treatment. The mainstream treatments for early‐phase Alzheimer's disease (AD) are acetylcholinesterase inhibitors (AChEIs); however, their efficacy and tolerability are unsatisfactory.^2^, ^3^ The unsatisfactory efficacy of AChEIs implies that there should be other mechanisms, such as oxidative stress and N‐methyl‐D‐aspartate receptor (NMDAR) dysfunction, contributing to the etiology of AD.^4^, ^5^

In addition to AChEIs, NMDAR antagonists have been developed to treat AD based on the ‘glutamate excitotoxicity theory’ for the middle‐late phase of AD.^6^ Meantime, a weak, partial, uncompetitive NMDAR antagonist, is approved for moderate–severe AD^7^; however, it has limited efficacy at the early phase.^8^ On the other hand, there is aging‐related reduction in glutamate content and synthesis in the cerebral cortex and hippocampus.^9^, ^10^ Further, the density of NMDARs declines with age^10^ and serum levels of D‐serine (an NMDAR co‐agonist) decrease in the patients with AD.^11^ NMDAR dysfunction may also lead to reduced antioxidant capacity, resulting in oxidative stress and in turn causing downregulation of NMDARs.^12^ Therefore, oxidative stress and NMDAR dysfunction interplay in the pathogenesis of AD.^4^, ^5^, ^12^

Sodium benzoate may play a neuroprotective role through reducing oxidative stress, based upon *in vitro* and *in vivo* evidence.^13^, ^14^ Benzoate may be also an indirect NMDAR enhancer, via inhibiting D‐amino acid oxidase (DAAO)^15^; it was shown to reverse NMDAR‐deficiency mediated animal behavior.^16^ Pilot studies found that benzoate was capable of improving cognitive function of patients with early‐phase AD,^17^ schizophrenia,^18^, ^19^ or late‐life depression,^20^ and altering brain activity (measured by functional magnetic resonance imaging [MRI]) in patients with mild cognitive impairment (MCI).^21^ In the pilot trial for early‐phase AD (*n* = 60),^17^ benzoate was given with a flexible dose strategy: the mean doses at weeks 8, 16, and 24 were 275.0 ± 76.3 (SD) mg/day, 525.0 ± 100.6 mg/day, and 716.7 ± 182.6 mg/day, respectively. However, the sample size has been modest, and the most efficacious doses and the prospective biomarkers (also for elucidating benzoate's underlying mechanisms) remain unknown.

The current study aimed to find out the optimal dose of benzoate for the treatment of AD in a larger sample of patients (*n* = 149) and to explore the prospective biomarkers. Glutathione (GSH), catalase (CAT), and superoxide dismutase (SOD) are three first‐line endogenous antioxidants, which prevent and reduce oxidative stress.^22^, ^23^ In addition, DAAO has been demonstrated to an NMDAR biomarker for cognitive aging in both cross‐sectional and longitudinal studies.^24^, ^25^ This study will check their potential for predicting treatment outcome.

## Methods

This 24‐week, randomized, double‐blind, placebo‐controlled trial was registered on the ClinicalTrials.gov (https://clinicaltrials.gov/ct2/show/NCT03752463), conducted by three major medical centers, Kaohsiung Chang Gung Memorial Hospital, Kaohsiung, Taiwan, China Medical University Hospital, Taichung, Taiwan, and Tsaotun Psychiatric Center, Nantou, Taiwan, and approved by the Institutional Review Board of Chang Gung Memorial Hospital, Taiwan (201405724A3C504), Institutional Review Board of China Medical University Hospital, Taiwan (CMUH104‐REC2‐090) and Institutional Review Board of Tsaotun Psychiatric Center, Taiwan (104039), and conducted in accordance with the current revision of the Declaration of Helsinki.

### Participants

One hundred and fifty‐four patients with AD were recruited. After a description of the study to the patients, written informed consents were obtained from all participants. Patients were evaluated by research psychiatrists after a thorough medical and neurological workup. Patients were enrolled into this study if they: (i) were aged 50–100; (ii) satisfied National Institute of Neurological and Communicative Disorders and Stroke and the AS and Related Disorders Association (NINCDS‐ADRDA)^26^ criteria for probable AD and had a clinical dementia rating (CDR)^27^ score of 1; (iii) were physically healthy and had all laboratory assessments (including blood routine, biochemical tests) within normal limits; (iv) had a mini‐mental state examination (MMSE)^28^ score of 10–26; and (v) had sufficient education to communicate effectively and are capable of following the protocol. For patients who had already been on AChEIs therapy, AChEIs had to be continued for at least 3 months before enrollment. AChEIs doses had to be kept unchanged during the study duration. For patients who had not yet been on AChEIs therapy, AChEIs or other anti‐dementia medications were forbidden during the study duration.

Exclusion criteria included: (i) Hachinski Ischemic Score > 4; (ii) substance abuse/dependence; (3) Parkinson disease, epilepsy, dementia with psychotic features; (iv) major depressive disorder; (v) major physical illnesses; and (vi) severe visual or hearing impairment.

### Study design

All eligible patients were randomly allocated to four groups: (i) benzoate 500 group (fixed dose at 500 mg/day); (ii) benzoate 750 group (500 mg/day for the first 4 weeks, 750 mg/day from the fifth week to the 24th week); (iii) benzoate 1000 group (500 mg/day for the first 4 weeks, 1000 mg/day from the fifth week to the 24th week); and (iv) placebo group. Two hundred and fifty milligrams of sodium benzoate or placebo were packed with identical capsules provided in coded containers. Patients were randomized through a computer‐generated randomization table to receive placebo or benzoate treatment in a 1:1:1:1 ratio. Non‐blinded pharmacists dispensed appropriate medication for treatment according to the randomization table. Patients, caregivers, and investigators, except the investigational pharmacist, were all blind to the assignment. Patient medical adherence and safety were closely monitored by caregivers and research physicians, and pill‐counting was monitored by the study staff.

During the study, limited use of benzodiazepines (up to 4‐mg/day lorazepam) was allowed as concomitant medication for agitation or insomnia. In case of side‐effect intolerance or clinical worsening, the patients could be withdrawn earlier.

### Clinical assessments

The primary outcome measure was the Alzheimer's Disease Assessment Scale‐cognitive subscale (ADAS‐cog)^29^ measured at weeks 0, 8, 16, and 24. The ADAS‐cog is the most popular and gold standard cognitive assessment instrument used in AD clinical trials.^30^ It consists of 11 tasks, including word recall, naming, commands, constructional praxis, ideational praxis, orientation, word recognition, instructions remembering, spoken language ability, word‐finding difficulty, and comprehension. Its scores range from 0 (best) to 70 (worst).

The secondary outcome measurements included Clinician's Interview‐Based Impression of Change plus Caregiver Input (CIBIC‐plus)^31^ for measuring global function at weeks 8, 16, and 24, and the composite score of a battery of additional cognitive tests measured at baseline (week 0) and endpoint (week 24).

The CIBIC‐plus is a global assessment of change based on a comprehensive, semi‐structured interview that includes caregiver‐supplied information. It is a seven‐point rating scale ranging from 1 to 7, where one represents markedly improved; four represents no change; and seven represents markedly worse.

The battery of additional cognitive tests included speed of processing (category fluency), working memory (Wechsler Memory Scale‐Third Edition, Spatial Span),^32^ and verbal learning and memory tests (Wechsler Memory Scale‐Third Edition, Word Listing).^32^ The cognition composite score was calculated by the average of the *T* scores of the three cognitive domains (speed of processing, working memory, and verbal learning and memory test). The raw score of each cognitive domain was standardized to a *T* score with a mean of 50 and a SD of 10 for making each test comparative. We have successfully utilized it in the previous study on early‐phase AD.^17^

Systemic adverse effects were evaluated every 8 weeks (at weeks 0, 8, 16, and 24) by physical and neurological examinations and laboratory tests including complete blood count (CBC) and biochemistry and reviewed by applying the Udvalg for Kliniske Undersogelser Side‐effects Rating Scale.^33^

Only raters who reached the intra‐class correlation coefficients of ≥0.90 during pre‐study training were allowed to rate the study patients. To maintain high inter‐rater reliability and to prevent rater drift, raters met at least once per quarter for training and reliability retesting. To minimize inter‐rater variability, each individual patient was assessed by the same raters throughout the trial.

### Laboratory measurements

DAAO and three antioxidants in peripheral blood were measured using the commercially available kits at baseline and endpoint.

DAAO protein levels in serum were assayed with the kit according to the manufacturer's recommended protocol (Cloud‐Clone Corp, Houston, TX, USA). The detailed method has been described elsewhere.^24^

GSH levels in plasma were measured using the kit according to the manufacturer's recommended protocol (Cayman, Ann Arbor, MI, USA). The detailed method has been described elsewhere.^34^

SOD levels in plasma were analyzed using the kit according to the manufacturer's recommended protocol (Merck, Kenilworth, NJ, USA). The detailed method has been described elsewhere.^35^

CAT in plasma was measured using the kit according to the manufacturer's recommended protocol (Cayman, Ann Arbor, MI, USA). The detailed method has been described elsewhere.^35^

All of the aforementioned analyses were repeated twice.

### Data analysis

Based on our previous trial in early‐phase AD (*n* = 30 in each group),^17^ in which the effect sizes of the primary and secondary outcome measures were between 0.73 and 0.86, it was estimated that the number of samples required for this study should have been sufficient to achieve a statistical power of 0.8 or more.

Chi‐square test (or Fisher's exact test) was used to compare differences of categorical variables and one‐way analysis of variance (or Kruskal–Wallis test if the distribution was not normal) for continuous variables among four treatment groups. The *t*‐test (or Mann–Whitney *U* test if the distribution was not normal) was applied to compare differences of continuous variables between groups. To compare the changes from baseline in repeated‐measure assessments, we used the generalized estimating equation (GEE) method's multiple linear regression models with treatment, visit, and treatment‐visit interaction terms after adjusting the baseline value of the outcome measure. The working correlation matrix was specified as autoregressive of order 1.

Subgroup analysis was also performed based on the previously reported gender difference (with female advantage) in benzoate treatment for the behavioral and psychological symptoms of dementia (BPSD).^36^

Multiple linear regression analyses were used to generate predictive models for treatment response.

All data were analyzed by SPSS version 22.0 (IBM Corp., Armonk, NY). All *P*‐values for clinical measures were based on two‐tailed tests with a significance level of 0.05.

## Results

### Patient disposition and characteristics

**Fig. 1:**
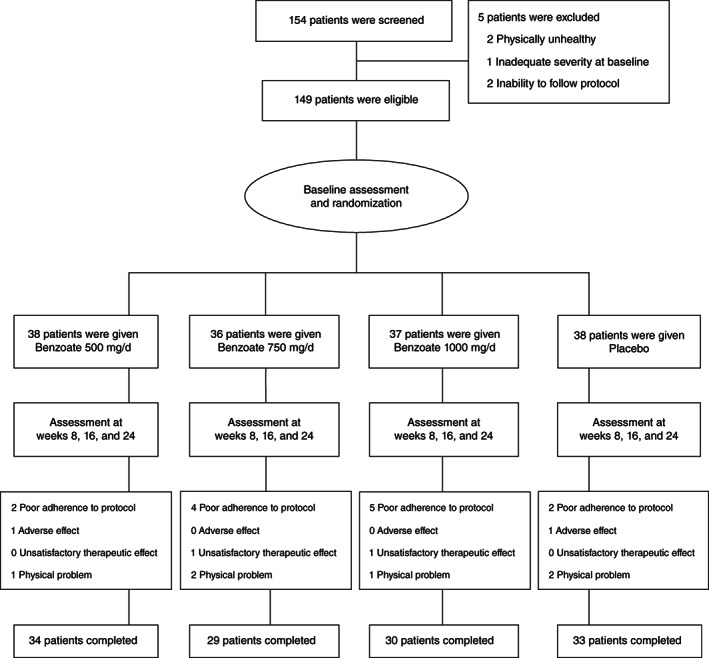
Flow diagram and disposition of the four treatment groups.

One hundred and fifty‐four patients were screened, and five of them were excluded due to being physically unhealthy (*n* = 2), inadequate severity at baseline (*n* = 1), and inability to follow protocol (*n* = 2). One hundred and forty nine patients were eligible and randomly allocated into four treatment groups (Fig. 1).

Of the 149 eligible patients, gender, age, education level, age at illness onset, illness duration, body mass index, MMSE, ADAS‐cog, and AChEI use (both percentage and dosage) at baseline were similar among the four groups (Table 1).

**Table 1:** Baseline clinical characteristics of the three benzoate groups (500, 750, and 1000 mg/day) and the placebo group

	Treatment Groups				
	BE500 (*n* = 38)	BE750 (*n* = 36)	BE1000 (*n* = 37)	Placebo (*n* = 38)	*P‐*value
Gender, female, *n* (%)	23 (60.5)	25 (69.4)	25 (67.6)	23 (60.5)	0.79^†^
Age, years, mean (SD)	75.2 (8.2)	73.8 (6.7)	74.2 (9.2)	75.8 (8.7)	0.52^‡^
Age at illness onset, years, mean (SD)	74.0 (8.5)	72.7 (7.1)	73.7 (8.8)	74.1 (8.8)	0.70^‡^
Illness duration, months, mean (SD)	17.3 (22.0)	15.6 (19.4)	11.1 (12.6)	19.6 (22.2)	0.65^‡^
Education, years, mean (SD)	5.2 (4.5)	5.9 (4.6)	5.2 (4.7)	5.3 (3.8)	0.89^‡^
BMI, mean (SD)	25.1 (5.0)	24.7 (3.8)	24.1 (4.8)	23.2 (3.6)	0.12^‡^
MMSE, mean (SD)	18.5 (5.5)	17.4 (5.8)	18.7 (5.8)	16.9 (7.1)	0.61^‡^
ADAS‐cog, mean (SD)	21.4 (10.0)	23.5 (12.9)	19.9 (9.3)	22.5 (13.2)	0.78^‡^
Patients using AChEIs					
Total, *n* (%)	12 (31.6)	13 (36.1)	8 (21.6)	15 (39.5)	0.38^†^
Donepezil, *n* (dose, mean ± SD)	6 (10.0 ± 0.0)	11 (9.5 ± 1.5)	4 (10.0 ± 0.0)	8 (8.8 ± 2.3)	0.40^‡^ ^,^ ^§^
Rivastigmine, *n* (dose, mean ± SD)	6 (8.5 ± 1.2)	2 (9.0 ± 0.0)	4 (9.0 ± 0.0)	7 (8.4 ± 1.7)	0.81^‡^ ^,^ ^§^
Galantamine, *n* (dose, mean ± SD)	0	0	0	0	

AChEIs, acetylcholine esterase inhibitors; ADAS‐cog, The Alzheimer's Disease assessment scale‐cognitive subscale; BE500, sodium benzoate 500 mg/day; BE750, sodium benzoate 750 mg/day; BE1000, sodium benzoate 1000 mg/day; BMI, body mass index; MMSE, mini‐mental state examination.

^†^ Chi‐square test.

^‡^ Kruskal–Wallis test.

^§^ Kruskal–Wallis test for the comparison of doses among four groups.

Finally, 34 (89.5%) patients in the benzoate 500 group, 29 (80.6%) in the benzoate 750 group, 30 (81.1%) in the benzoate 1000 group, and 33 (86.8%) in the placebo group completed the 24‐week trial (Fig. 1). The most frequent reason of early withdrawal was poor adherence to protocol (*n* = 2 in the benzoate 500 group, four in the benzoate 750 group, five in the benzoate 1000 group, and two in the placebo group) (Fig. 1). The other reasons included adverse effects (*n* = 1 in the benzoate 500 group, and one in the placebo group), unsatisfactory therapeutic response (*n* = 1 in the benzoate 750 group, and one in the benzoate 1000 group), and physical problems (*n* = 1 in the benzoate 500 group, two in the benzoate 750 group, one in the benzoate 1000 group, and two in the placebo group) (Fig. 1).

### Primary outcome measures

Compared to the placebo group, the benzoate 1000 group performed best in improving ADAS‐cog performance (that is, reducing ADAS‐cog score; *P* = 0.026 and 0.029 at week 24 and endpoint, respectively), while the benzoate 750 group also showed improvement in ADAS‐cog (*P* = 0.042 and 0.043 at week 24 and endpoint, respectively) (Table 2). The benzoate 500 mg/day and the placebo did not differ significantly in altering ADAS‐cog at all visits and endpoint (Table 2).

**Table 2:** Results of measures of primary outcome over the 24‐week treatment using generalized estimating equations (GEE) Method, which simultaneously compared the four treatment groups using a single analysis

Scale	BE500	BE750	BE1000	Placebo	BE500 vs placebo	BE750 vs placebo	BE1000 vs placebo
ADAS‐cog	Mean ± SD (*N*)	Mean ± SD (*N*)	Mean ± SD (*N*)	Mean ± SD (*N*)	Estimate, SE, *Z* (*P* value)	Estimate, SE, *Z* (*P* value)	Estimate, SE, *Z* (*P* value)
Baseline	21.4 ± 10.0 (38)	23.5 ± 12.9 (35)	19.9 ± 9.3 (37)	22.5 ± 13.2 (38)	−1.14, 2.65, −0.43 (0.67)^†^	0.99, 3.01, 0.33 (0.74)^†^	−2.64, 2.59, −1.02 (0.31)^†^
Week 8	19.7 ± 9.2 (36)	21.5 ± 13.2 (33)	18.4 ± 10.3 (34)	22.1 ± 15.9 (34)	−1.52, 1.30, −1.16 (0.25)^‡^	−2.54, 1.47, −1.73 (0.08)^‡^	−1.68, 1.27, −1.32 (0.19)^‡^
Week 16	19.0 ± 10.0 (34)	20.1 ± 13.2 (32)	17.3 ± 10.7 (33)	20.6 ± 15.6 (33)	−1.35, 1.65, −0.82 (0.41)^‡^	−2.74, 1.82, −1.51 (0.13)^‡^	−1.98, 1.58, −1.25 (0.21)^‡^
Week 24	20.2 ± 10.8 (34)	21.1 ± 13.6 (29)	16.8 ± 10.6 (30)	21.2 ± 14.6 (33)	−0.97, 1.50, −0.65 (0.52)^‡^	−2.89, 1.42, −2.03 (0.042)^‡^	−2.84, 1.28, −2.22 (0.026)^‡^
Endpoint	20.4 ± 11.2 (36)	20.9 ± 13.6 (33)	17.2 ± 10.5 (34)	22.3 ± 15.6 (34)	−0.97, 1.50, −0.65 (0.52)^‡^	−2.89, 1.43, −2.02 (0.043)^‡^	−2.79, 1.28, −2.18 (0.029)^‡^

ADAS‐cog, The Alzheimer's Disease Assessment Scale‐Cognitive Subscale; BE500, sodium benzoate 500 mg/day; BE750, sodium benzoate 750 mg/day; BE1000, sodium benzoate 1000 mg/day; SE, standard error.

^†^ Comparison was based on the average of the total score.

^‡^ Comparisons was based on the changes from the baseline in average of total score. Estimate is the coefficient of treatment and treatment‐visit interaction term in the GEE method's multiple linear regression model by specifying the working correlation matrix as autoregressive of order 1, AR (1). *P*‐values were based on two‐tailed tests.

**Fig. 2:**
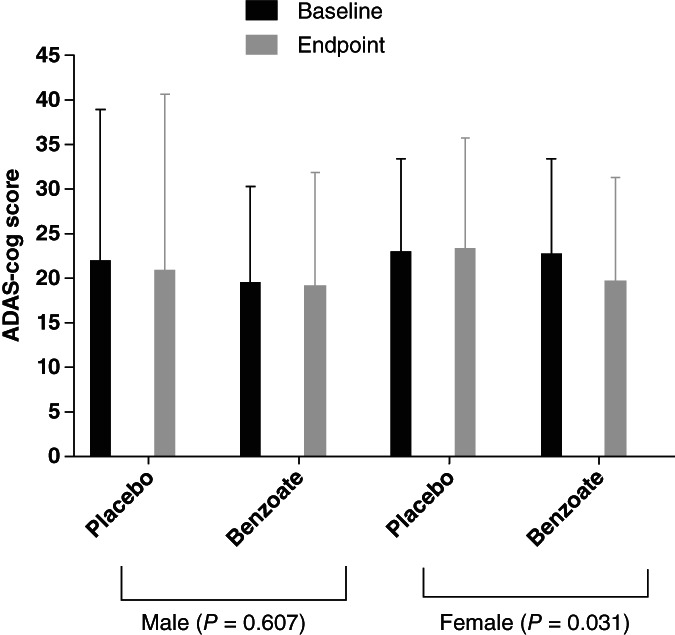
Gender difference after 24‐week treatment of sodium benzoate with 500, 750 mg/day, or 1000 mg/day. Women but not men showed significant improvement in cognitive function, measured by Alzheimer's disease assessment scale‐cognitive subscale (ADAS‐cog), after benzoate treatment. Among the 96 women, benzoate surpassed placebo in the effect on ADAS‐cog (*t* = −2.196; *P* = 0.031). Among the 53 men, benzoate and placebo treatments did not differ significantly in ADAS‐cog.

As shown in Figure 2, women appeared more likely to improve in the ADAS‐cog performance than men after benzoate treatment. Regarding the dose difference among the male patients, the benzoate 1000 group, but not the other two benzoate groups, showed a trend (insignificant) improvement in the ADAS‐cog performance than the placebo group at week 24 (*P* = 0.10) and endpoint (*P* = 0.11) (not shown). Of note, the sample size in men (with 15 participants in the placebo group, 15 in the benzoate 500 group, 11 in the benzoate 750 group, and 12 in the benzoate 1000 group) was inadequate. Therefore, the subgroup analyses focused on the female group (with 23 participants in the placebo group, 23 in the benzoate 500 group, 25 in the benzoate 750 group, and 25 in the benzoate 1000 group).

Regarding the dose difference among the female patients, the benzoate 750 group, compared to the placebo group, improved significantly in ADAS‐cog at week 8 (estimate = −4.03, SE = 1.80, *Z* = −2.24, *P* = 0.025), week 16 (estimate = −5.37, SE = 2.35, *Z* = −2.29, *P* = 0.022), week 24 (estimate = −5.05, SE = 1.84, *Z* = −2.75, *P* = 0.006), and endpoint (estimate = −5.07, SE = 1.84, *Z* = −2.76, *P* = 0.006); the benzoate 1000 group displayed a trend (insignificant) improvement in ADAS‐cog at week 24 (*P* = 0.086) and endpoint (*P* = 0.082); and the benzoate 500 group did not differ significantly from the placebo group in improving ADAS‐cog at each visit (not shown).

### Secondary outcome measures

As shown in Table S1, the four treatment groups did not differ significantly in changing the performances in CIBIC‐plus and the additional cognition composite.

The subgroup analysis for the secondary outcome measures focused on the female group, because, as aforementioned, the sample size of the male group appeared too small. Among the female patients, the benzoate 750 group excelled the placebo group in CIBIC‐plus at endpoint (estimate = −0.38, SE = 0.20, *Z* = −1.97, *P* = 0.049). The benzoate 1000 group showed a trend (insignificant) superiority over the placebo group in CIBIC‐plus at endpoint (estimate = −0.33, SE = 0.21, *Z* = −1.55, *P* = 0.12). The benzoate 500 and the placebo groups were similar in CIBIC‐plus performance (not shown).

Among the female patients, the four treatment groups did not differ significantly in changing the performances in the additional cognition composite (not shown).

### Adverse effects

The four treatment groups showed similar performances in adverse effects, as measured by Udvalg for Kliniske Undersøgelser (UKU) Side‐effects Rating Scale^33^ (Table S2). The total frequency of treatment‐emergent adverse events was 13 patients in the benzoate 500 group, 12 in the benzoate 750 group, eight in the benzoate 1000 group, and 13 in the placebo group (Table S2).

### Blood levels of DAAO and antioxidants

All laboratory parameters were measured at baseline and endpoint (Table S3). At baseline, the four treatment groups had similar blood levels of DAAO, GSH, SOD, and CAT (Table S3).

After treatment, the difference in plasma CAT among the four treatment groups did not reach significance (*P* = 0.07, Table S3). However, plasma CAT tended to increase in all benzoate receivers as a whole, when compared to the placebo recipients (*P* = 0.29, Table 3).

**Table 3:** Measures of DAAO and antioxidants over the 24‐week treatment between the benzoate and placebo recipients

Measure	Benzoate	Placebo	*P*‐value
DAAO (ng/mL)	Mean ± SD (*N*)	Mean ± SD (*N*)	
Baseline	49.0 ± 11.0 (92)	52.9 ± 10.4 (32)	
Endpoint	48.3 ± 11.0 (78)	50.9 ± 7.7 (27)	
Difference	−1.2 ± 5.9 (78)	−3.5 ± 8.2 (27)	0.12^†^
GSH (uM)	Mean ± SD (*N*)	Mean ± SD (*N*)	
Baseline	7.6 ± 4.5 (92)	6.4 ± 4.4 (32)	
Endpoint	7.3 ± 4.4 (78)	4.9 ± 3.4 (27)	
Difference	−0.2 ± 3.8 (78)	−1.2 ± 2.1 (27)	0.095^†^
SOD (U/mL)	Mean ± SD (*N*)	Mean ± SD (*N*)	
Baseline	0.02 ± 0.09 (92)	0.04 ± 0.10 (32)	
Endpoint	0.03 ± 0.09 (77)	0.06 ± 0.10 (27)	
Difference	0.00 ± 0.07 (77)	0.02 ± 0.08 (27)	0.40^‡^
CAT (nmol/min/mL)	Mean ± SD (*N*)	Mean ± SD (*N*)	
Baseline	56.9 ± 27.5 (92)	54.2 ± 26.3 (32)	
Endpoint	58.5 ± 24.7 (78)	46.8 ± 21.7 (27)	
Difference	1.8 ± 27.2 (78)	−4.8 ± 28.4 (27)	0.29^†^

CAT, catalase; DAAO, D‐amino acid oxidase; GSH, glutathione; SD, standard deviation; SOD, superoxide dismutase.

^†^ *t* test.

^‡^ Mann–Whitney *U* test.

GSH tended to decrease less in benzoate‐treated patients, when compared to placebo recipients (*P* = 0.095, Table 3).

The benzoate recipients and the placebo group did not differ significantly in DAAO and SOD levels after treatment (Table 3).

### Predictive biomarkers

Multiple linear regression analyses were used to generate predictive models for cognitive outcome in the ADAS‐cog (the higher score meaning a worse outcome) among the benzoate recipients (Table 4).

**Table 4:** Multiple linear regression analyses (backward) of independent factors associated with ADAS‐cog improvements (score reduction from baseline to endpoint) in sodium benzoate group

ADAS‐cog improvement			
Variable	B (SE)	*t*	*P*
Gender, female vs male	3.646 (1.417)	2.573	0.012
Baseline CAT level	0.081 (0.024)	3.380	0.001
Adjusted *R* ^2^ = 0.152			

The variables were demographic and clinical characteristics including gender, age, age at illness onset, education, BMI, anti‐dementia medication use, benzoate dose, baseline ADAS‐cog score, and laboratory parameters at baseline including DAAO, and antioxidants (GSH, SOD and CAT). Significant variables are shown in the Table (*P* < 0.05).

ADAS‐cog, Alzheimer's disease assessment scale‐cognitive subscale; BMI, body mass index; CAT, catalase; DAAO, D‐amino acid oxidase; GSH, glutathione; SOD, superoxide dismutase.

Baseline plasma CAT was correlated with ADAS‐cog improvement after treatment (*P* = 0.001, Table 4). That is, higher baseline CAT predicted better cognitive outcome after treatment. In addition, the female patients excelled the male patients in improving cognitive function after 24‐week treatment (*P* = 0.012, Table 4).

Moreover, differences in the level of CAT were correlated with the magnitudes of ADAS‐cog improvement (score reduction from baseline to endpoint) (*r* = 0.25; *P* = 0.029) in the benzoate‐treated group, not in the placebo group.

## Discussion

The main findings of this benzoate dose‐finding, randomized, double‐blind, placebo‐controlled trial for AD included: (i) 1000 mg of benzoate led to the best outcome, while 750 mg/day also generated similar efficacy to 1000 mg/day (Table 2); (ii) women performed better than men (Fig. 2, Table 4); (iii) higher CAT in plasma before treatment predicted better cognitive outcome after treatment (Table 4); and (iv) benzoate‐treated patients tended to have higher CAT and GSH than placebo‐receivers after treatment (Table 3). Moreover, in accordance with the previous pilot, small‐sized study (*n* = 60),^17^ the current study (*n* = 149) further confirmed benzoate as a novel therapeutic approach for AD.

In addition to a potential DAAO inhibitor,^15^ sodium benzoate may also diminish oxidative stress.^13^, ^14^ GSH, CAT, and SOD are three first‐line endogenous antioxidants, which prevent and reduce oxidative stress.^22^, ^23^ We therefore selected these four candidate biomarkers (DAAO, GSH, CAT, and SOD) for this trial.

Blood DAAO levels were found to be related with cognitive aging in both cross‐section and prospective studies.^24^, ^25^ In the current study, benzoate treatment at any dose did not differ from placebo in altering blood DAAO levels. Albeit a DAAO inhibitor *in vitro*,^15^ sodium benzoate did not display DAAO inhibitory activity while it indeed generated antipsychotic effects in the phencyclidine animal model of schizophrenia.^16^ Whether benzoate treatment can change DAAO in human CSF or brain requires further studies.

Supporting the oxidative stress theory of AD,^4^, ^5^ benzoate treatment rescued cognition of AD patients *via* augmenting two vital endogenous antioxidants, CAT and GSH. Earlier, benzoate was also able to increase plasma CAT in treatment‐resistant schizophrenia patients; importantly, the CAT increment was positively related with clinical improvement.^37^ However, benzoate at any dose was unable to change SOD levels, when compared to placebo. Correspondingly, benzoate 1000 mg/day, benzoate 2000 mg/day, and placebo treatment displayed similar effects on blood SOD levels in treatment‐resistant schizophrenia patients.^37^ While reductions of CAT, GSH, and SOD have been implicated in pathogenesis of AD,^38^, ^39^, ^40^, ^41^ the decrease of SOD has been inconsistent.^42^ A compensatory mechanism of SOD may be involved.^23^ In accordance, only blood GSH and CAT were altered in the current study.

Sodium benzoate's oxidative‐stress reduction actitivity^13^, ^14^ was found to be higher in women, especially under a medium dose, rather than a high dose.^43^ Women were more likely to respond to benzoate treatment than men in a recent clinical trial for BPSD.^36^ Benzoate also increased estradiol to follicle‐stimulating hormone (FSH) ratios among women with BPSD.^36^ Of note, in a recent study,^44^ blocking FSH action improved cognitive function of mice with AD. Furthermore, in the present study, benzoate also yielded better outcomes in women, with the best efficacy under 750 mg/day, rather than 1000 mg/day. In contrast, among the male patients, 1000 mg/day appeared to be the best; however, the same size was too small to be conclusive. Future larger‐sized studies, with various doses (including doses higher than 1000 mg), are warranted for the male patients.

For detailed comparisons, in the previous pilot AD trial,^17^ benzoate treatment improved the primary outcome (ADAS‐cog) and both of the secondary outcomes (CIBIC‐plus and additional cognitive composite). In the current trial, (benzoate improved ADAS‐cog) in all subjects and CIBIC‐plus in women, but not additional cognition composite. The main reason for the discrepancy may be that benzoate‐receivers in the previous study^17^ were younger in age than those in the present study (mean = 70.7 vs 73.8 years [750‐mg/day receivers] or 74.2 years [1000‐mg/day]). It has been well known that early detection and treatment is essential for the outcome of Alzheimer's disease.^45^ In addition, the different findings between the primary and the secondary outcomes also support the notion that the ADAS‐cog is the gold standard for the AD clinical trials.^30^

In terms of the safety domain, the current study found that all three doses (500, 750, and 1000 mg/day) of benzoate and placebo had similar performance (Table S2). In accordance, the previous studies using flexible‐dose sodium benzoate also showed a similar safety profile with that of placebo in early‐phase AD (250–750 mg/day),^17^ late‐life depression (250–1500 mg/day),^20^ MCI (250–1500 mg/day),^21^ and BPSD (250–1500 mg/day).^36^

This study was limited by its moderate sample size, which might have led to underpowered results. Further, regarding multiplicity adjustments, this study had one primary outcome (ADAS‐cog) and two secondary outcomes (CIBIC‐plus and additional cognition composite). If we considered the primary and secondary outcomes separately, it would have been dispensable to correct the *P*‐value for the primary outcome but necessary to correct the *P*‐values for the secondary outcomes (which, however, had been insignificant even without correcting). If we took all outcomes simultaneously, the significancy of the primary outcome would have become insignificant after correcting the *P*‐value. Therefore, future clinical trials with larger sample sizes are needed. The second limitation is the 24‐week treatment duration. It remains unclear whether a longer‐term (such as 48 weeks) treatment of benzoate can improve the cognitive and general function. Thirdly, whether the finding in Han Taiwanese can be extrapolated to other populations requires further studies. Fourthly, we did not measure DAAO, D‐serine, and the three antioxidants in the brain, and the blood–brain relationships of these potential biomarkers remain unknown in the participants. Moreover, a recent study^21^ demonstrated that sodium benzoate was able to alter brain activity (measured by resting‐state functional MRI) as well as cognitive functions in individuals with MCI. Biomarkers such as cerebrospinal fluid and neuroimaging data may also enhance the measurement methodology in future AD studies.

## Conclusions

This study suggests that sodium benzoate, *via* augmenting two vital endogenous antioxidants, CAT and GSH, shows promise as a novel treatment for AD. Women and the patients with higher plasma CAT before treatment were associated with better treatment outcome. If these findings can be reconfirmed in future larger‐sized studies in other populations, this innovative approach for AD will instill hope for the rapidly growing dementia population.

## Disclosure statement

All authors report no biomedical financial interests or potential conflicts of interest.

## Author contributions

C.‐H.L. had full access to all of the data in the study and takes responsibility for the integrity of the data and the accuracy of the data analysis. Concept and design: C.‐H.L., H.‐Y.L. Acquisition, analysis, or interpretation of data: C.‐H.L., S.‐H.W., H.‐Y.L. Drafting of the manuscript: C.‐H.L., H.‐Y.L. Statistical analysis: C.‐H.L., S.‐H.W. Obtained funding: C.‐H.L., H.‐Y.L. Administrative, technical, or material support: C.‐H.L., H.‐Y.L. Supervision: C.‐H.L., H.‐Y.L.

## Funding statement

This work was supported by the National Health Research Institutes, Taiwan (NHRI‐EX110‐10816NC; NHRI‐EX111‐11133NI); the Ministry of Science and Technology in Taiwan (MOST 109‐2628‐B‐182A‐002; 110‐2314‐B‐182A‐048‐; MOST 109‐2314‐B‐039‐039‐MY3); the Chang Gung Memorial Hospital, Taiwan (CMRPG8G1391, CMRPG8K1162, CMRPG8K1462), and the China Medical University Hospital, Taiwan (DMR‐HHC‐111‐9). The sponsors were not involved in the design and conduct of the study; collection, management, analysis, and interpretation of the data; and preparation, review, or approval of the manuscript.

## Ethics statement

This study was approved by the Institutional Review Board of Chang Gung Memorial Hospital, Taiwan (201405724A3C504), Institutional Review Board of China Medical University Hospital, Taiwan (CMUH104‐REC2‐090) and Institutional Review Board of Tsaotun Psychiatric Center, Taiwan (104039), and conducted in accordance with the current revision of the Declaration of Helsinki.

## Supporting information


**Table S1.** Results of measures of secondary outcomes over the 24‐week treatment using generalized estimating equations (GEE) method, which simultaneously compared the four treatment groups using a single analysis.
**Table S2.** Treatment‐emergent adverse events during the study.
**Table S3.** Measures of DAAO and antioxidants over the 24‐week treatment of the four treatment groups.

## Data Availability

Data available: No. Explanation for why data not available: The data will be available under request approved by IRB.
