# Supplementary material for: Endogenous antioxidants predicted outcome and increased after treatment: A benzoate dose‐finding, randomized, double‐blind, placebo‐controlled trial for Alzheimer's disease
Source: Psychiatry Clin Neurosci. 2022 Nov 24;77(2):102–9. doi: 10.1111/pcn.13504 (PMC10099492; doi:10.1111/pcn.13504)
Supplement: Supplementary file 1 — Table S1. Results of measures of secondary outcomes over the 24‐week treatment using generalized estimating equations (GEE) method, which simultaneously compared the four treatment groups using a single analysis. Table S2. Treatment‐emergent adverse events during the study. Table S3. Measures of DAAO and antioxidants over the 24‐week treatment of the four treatment groups. [file PCN-77-102-s001.docx]

**Supplementary Table S1. Results of measures of secondary outcomes over the 24-week treatment using generalized estimating equations (GEE) method, which simultaneously compared the four treatment groups using a single analysis**

| **Scale** | **BE500** | **BE750** | **BE1000** | **Placebo** | **BE500 vs. placebo** | **BE750 vs. placebo** | **BE1000 vs. placebo** |
| --- | --- | --- | --- | --- | --- | --- | --- |
| CIBIC-plus | **Mean ± SD (N)** | **Mean ± SD (N)** | **Mean ± SD (N)** | **Mean ± SD (N)** | **Estimate, SE, Z (*P* value)** | **Estimate, SE, Z (*P* value)** | **Estimate, SE, Z (*P* value)** |
| Week 8 | 3.72 ± 0.66 (36) | 3.67 ± 0.69 (33) | 3.65 ± 0.60 (34) | 3.50 ± 0.62 (34) | 0.22, 0.15, 1.48 (.14)^b^ | 0.17, 0.16, 1.06 (.29)^b^ | 0.15, 0.14, 1.02 (.31)^a^ |
| Week 16 | 3.76 ± 0.86 (34) | 3.63 ± 0.71 (32) | 3.76 ± 0.61 (33) | 3.52 ± 0.67 (33) | 0.25, 0.18, 1.37 (.17)^b^ | 0.16, 0.17, 0.96 (.34)^b^ | 0.27, 0.15, 1.73 (.08)^b^ |
| Week 24 | 3.91 ± 0.87 (34) | 3.62 ± 0.56 (29) | 3.57 ± 0.57 (30) | 3.73 ± 0.72 (33) | 0.18, 0.19, 0.98 (.33)^b^ | -0.05, 0.16, -0.29 (.77)^b^ | -0.08, 0.16, -0.49 (.63)^b^ |
| Endpoint | 3.89 ± 0.85 (36) | 3.67 ± 0.60 (33) | 3.65 ± 0.60 (34) | 3.71 ± 0.72 (34) | 0.18, 0.19, 0.99 (.32)^b^ | -0.04, 0.16, -0.25 (.80)^b^ | -0.06, 0.16, -0.37 (.71)^b^ |
| Cognition† | **Mean ± SD (N)** | **Mean ± SD (N)** | **Mean ± SD (N)** | **Mean ± SD (N)** | **Estimate, SE, Z (*P* value)** | **Estimate, SE, Z (*P* value)** | **Estimate, SE, Z (*P* value)** |
| Baseline | 49.6 ± 6.6 (38) | 49.4 ± 5.6 (35) | 51.4 ± 7.9 (37) | 49.5 ± 7.9 (38) | 0.09, 1.65, 0.05 (.96)^a^ | -0.14, 1.58, -0.09 (.93)^a^ | 1.87, 1.81, 1.03 (.30)^a^ |
| Endpoint | 49.2 ± 6.4 (34) | 49.5 ± 5.9 (31) | 51.1 ± 7.7 (31) | 49.7 ± 9.0 (33) | -0.67, 1.27, -0.53 (.60)^b^ | -0.09, 1.17, -0.08 (.94)^b^ | -0.48, 1.13, -0.42 (.67)^b^ |

Abbreviations: BE500, sodium benzoate 500 mg/day; BE750, sodium benzoate 750 mg/day; BE1000, sodium benzoate 1000 mg/day; SE, standard error;

CIBIC-plus, Clinician's Interview-Based Impression of Change plus Caregiver Input.

^a^ Comparison was based on the average of the total score. ^b^ Comparisons was based on the changes from the baseline in average of total score. Estimate is the coefficient of treatment and treatment-visit interaction term in the GEE method’s multiple linear regression model by specifying the working correlation matrix as autoregressive of order 1, AR(1). P values were based on two-tailed tests.

† Additional cognition composite, the composite test score of speed of processing, working memory, and verbal learning, and memory.

**Supplementary Table S2. Treatment-emergent Adverse Events during the Study**

|  | **No. of Subjects** | | | | | | | |
| --- | --- | --- | --- | --- | --- | --- | --- | --- |
|  | | **BE500** | | **BE750** | | **BE1000** | | **Placebo** |
| Asthenia/Increased Fatigability | 2 | |  | |  | |  | |
| Failing Memory | 2 | | 3 | | 1 | | 2 | |
| Depression |  | | 1 | |  | | 2 | |
| Tension/Inner Unrest |  | |  | |  | | 1 | |
| Increased Duration of Sleep |  | |  | |  | | 1 | |
| Reduced Duration of Sleep | 3 | | 3 | | 1 | | 2 | |
| Dystonia |  | | 1 | |  | | 1 | |
| Akathisia |  | |  | |  | | 1 | |
| Nausea/Vomiting | 2 | | 1 | |  | |  | |
| Diarrhoea |  | |  | | 2 | |  | |
| Constipation | 1 | |  | |  | |  | |
| Polyuria/Polydipsia | 1 | |  | |  | |  | |
| Orthostatic Dizziness | 2 | | 2 | | 3 | | 3 | |
| Weight Loss |  | |  | | 1 | |  | |
| Headache, Tension Headache |  | | 1 | |  | |  | |
| Total | 13 | | 12 | | 8 | | 13 | |

Abbreviation: BE500, sodium benzoate 500 mg/day; BE750, sodium benzoate 750 mg/day;

BE1000, sodium benzoate 1000 mg/day.

**Supplementary Table S3. Measures of DAAO and Antioxidants over the 24-Week Treatment of the Four Treatment Groups**

| **Measure** | **BE500** | **BE750** | **BE1000** | **Placebo** | ***P* value** |
| --- | --- | --- | --- | --- | --- |
| DAAO, ng/mL | **Mean ± SD (N)** | **Mean ± SD (N)** | **Mean ± SD (N)** | **Mean ± SD (N)** |  |
| Baseline | 51.7 ± 11.2 (32) | 46.6 ± 12.4 (29) | 48.5 ± 9.0 (31) | 52.9 ± 10.4 (32) | .09 ^a^ |
| Endpoint | 48.4 ± 12.1 (28) | 47.7 ± 11.4 (24) | 48.8 ± 9.6 (26) | 50.9 ± 7.7 (27) | .71 ^a^ |
| GSH, uM | **Mean ± SD (N)** | **Mean ± SD (N)** | **Mean ± SD (N)** | **Mean ± SD (N)** |  |
| Baseline | 7.0 ± 4.2 (32) | 7.8 ± 4.5 (29) | 8.0 ± 4.8 (31) | 6.4 ± 4.4 (32) | .47 ^b^ |
| Endpoint | 7.2 ± 4.0 (28) | 8.0 ± 4.9 (24) | 6.8 ± 4.4 (26) | 4.9 ± 3.4 (27) | .07 ^b^ |
| SOD, U/mL | **Mean ± SD (N)** | **Mean ± SD (N)** | **Mean ± SD (N)** | **Mean ± SD (N)** |  |
| Baseline | 0.02 ± 0.06 (32) | 0.04 ± 0.11 (29) | 0.01 ± 0.09 (31) | 0.04 ± 0.10 (32) | .89 ^b^ |
| Endpoint | 0.03 ± 0.08 (27) | 0.03 ± 0.09 (24) | 0.02 ± 0.09 (26) | 0.06 ± 0.10 (27) | .44 ^a^ |
| CAT, nmol/min/mL | **Mean ± SD (N)** | **Mean ± SD (N)** | **Mean ± SD (N)** | **Mean ± SD (N)** |  |
| Baseline | 54.7 ± 27.4 (32) | 58.9 ± 31.7 (29) | 57.4 ± 23.7 (31) | 54.2 ± 26.3 (32) | .90 ^a^ |
| Endpoint | 57.7 ± 26.6 (28) | 60.0 ± 26.1 (24) | 58.1 ± 22.1 (26) | 46.8 ± 21.7 (27) | .19 ^a^ |

Abbreviations: DAAO, D-amino acid oxidase; GSH, glutathione; SOD, superoxide dismutase; CAT, catalase; SD, standard deviation.

^a^ ANOVA test; ^b^ Kruskal Wallis test
